# Genetic divergence of HIV-1 B subtype in Italy over the years 2003–2016 and impact on CTL escape prevalence

**DOI:** 10.1038/s41598-018-34058-7

**Published:** 2018-10-24

**Authors:** Claudia Alteri, Lavinia Fabeni, Rossana Scutari, Giulia Berno, Domenico Di Carlo, Caterina Gori, Ada Bertoli, Alessandra Vergori, Ilaria Mastrorosa, Rita Bellagamba, Cristina Mussini, Manuela Colafigli, Francesco Montella, Alfredo Pennica, Claudio Maria Mastroianni, Enrico Girardi, Massimo Andreoni, Andrea Antinori, Valentina Svicher, Francesca Ceccherini-Silberstein, Carlo Federico Perno, Maria Mercedes Santoro

**Affiliations:** 1 Department of Experimental Medicine and Surgery, University of Rome “Tor Vergata”, Rome, 00133 Italy; 2 National Institute for Infectious Diseases L. Spallanzani, IRCCS, Rome, 00161 Italy; 3 Department of Biomedical and Clinical Sciences “L. Sacco”, University of Milan, Pediatric Clinical Research Center “Romeo and Enrica Invernizzi”, Milan, 20133 Italy; 4 University Hospital of Modena, Modena, 41121 Italy; 5 San Gallicano Hospital, IFO-IRCCS, Rome, 00144 Italy; 6 San Giovanni Hospital, Rome, 00184 Italy; 7 Sant’Andrea Hospital, Rome, 00189 Italy; 8 University of Rome “La Sapienza”, Rome, 00185 Italy; 9 University Hospital of Rome “Tor Vergata”, Rome, 00133 Italy; 10 Department of Oncology, University of Milan, Milan, 20122 Italy

## Abstract

HIV-1 is characterized by high genetic variability, with implications for spread, and immune-escape selection. Here, the genetic modification of HIV-1 B subtype over time was evaluated on 3,328 *pol* and 1,152 *V3* sequences belonging to B subtype and collected from individuals diagnosed in Italy between 2003 and 2016. Sequences were analyzed for genetic-distance from consensus-B (Tajima-Nei), non-synonymous and synonymous rates (dN and dS), CTL escapes, and intra-host evolution over four time-spans (2003–2006, 2007–2009, 2010–2012, 2013–2016). Genetic-distance increased over time for both *pol* and *V3* sequences (P < 0.0001 and 0.0003). Similar results were obtained for dN and dS. Entropy-value significantly increased at 16 *pol* and two *V3* amino acid positions. Seven of them were CTL escape positions (protease: 71; reverse-transcriptase: 35, 162, 177, 202, 207, 211). Sequences with ≥3 CTL escapes increased from 36.1% in 2003–2006 to 54.0% in 2013–2016 (P < 0.0001), and showed better intra-host adaptation than those containing ≤2 CTL escapes (intra-host evolution: 3.0 × 10^−3^ [2.9 × 10^−3^–3.1 × 10^−3^] vs. 4.3 × 10^−3^ [4.0 × 10^−3^–5.0 × 10^−3^], P[LRT] < 0.0001[21.09]). These data provide evidence of still ongoing modifications, involving CTL escape mutations, in circulating HIV-1 B subtype in Italy. These modifications might affect the process of HIV-1 adaptation to the host, as suggested by the slow intra-host evolution characterizing viruses with a high number of CTL escapes.

## Introduction

Human immunodeficiency virus type 1 (HIV-1) is characterized by a high genetic variability, mainly due to high rate of replication, mutation, and recombination^1–3^. Amino acid mutations that arise by chance can be thereafter selected by the pressure of the human immune system^4,5^ or combined antiretroviral therapy (cART)^6,7^. New selected amino acids may establish strong interactions among themselves, further modulating viral fitness, and development of drug resistance, but also immune escape process, thus enhancing pathogenic potential of the virus^8,9^.

Moreover, due to the continuum intent of HIV to find the best host adaptation, a constant selection of new viral species can occur at the intra-host level^10^. If these viral variants enhance viral competitive ability, they will rapidly spread at the epidemiological level becoming dominant variants characterized by selective advantage^11,12^.

To date, literature on HIV-1 evolutionary dynamics focused attention on the spread of drug resistance and on the potential that drug resistant mutations, together with compensatory mutations, had on increased fitness and virulence in patients never exposed to or starting cART^13–15^. Much has also been done in the field of CD8+ cytotoxic T-lymphocyte (CTL) related regions, their intra-host evolution and their relationship to clinical outcome^16–18^.

On the other hand, little is known regarding the extent of HIV-1 genetic modification over time, and regarding the role that CTL-mediated selection pressure has in inter-host HIV-1 evolution, even if some evidence has recently emerged^19,20^. Moreover, if and how fixation of CTL escapes may influence viral load and immunological parameters in the first phases of infection is still largely unknown.

Thus, in the present study, we investigated the extent of genetic divergence, CTL escape prevalence and intra-host evolution of the most prevalent HIV-1 subtype, the B form, in a cohort of HIV-1 infected patients diagnosed in Italy between 2003 and 2016.

## Results

### Study population

A total of 3,328 HIV-1 B subtype infected individuals with at least one polymerase (*pol)* sequence at HIV-1 diagnosis (range of weeks between HIV-1 diagnosis and *pol* sequencing: 0.8–5.0) were retrieved for this study. *V3* sequences collected at the same time point of *pol* were also available for a subgroup of patients (N = 1,152).

By dividing the population in accordance with year of HIV-1 diagnosis, 554 individuals belonged to the time frame 2003–2006, 826 to 2007–2009, 1,003 to 2010–2012, and 945 to 2013–2016 (Table 1).

**Table 1:** Patients’ characteristic.

Characteristic	Overall	2003–2006	2007–2009	2010–2012	2013–2016	P-value^a^
Number, n	3,328	554	826	1,003	945	
Male, n (%)	2,814 (84.6)	443 (80.0)	696 (84.3)	875 (87.2)	800 (84.7)	<0.0001
Year of first GRT, median (IQR)	2010 (2008–2013)	2005 (2004–2006)	2008 (2007–2009)	2011 (2010–2012)	2014 (2013–2015)	—
Year of diagnosis, median (IQR)	2010 (2007–2013)	2005 (2004–2006)	2008 (2007–2009)	2011 (2010–2012)	2014 (2013–2015)	—
Age at GRT, median (IQR)	38 (31–46)	38 (32–45)	38 (31–45)	38 (31–46)	39 (31–48)	0.358
Plasma viral load at GRT, (copies/mL), median (IQR)	76,619 (21,216-258,222)	82,285 (20,191–300,000)	64,958 (19,200-224,161)	81,421 (21,269-266,060)	78,434 (25,280-255,374)	0.131
CD4+T at GRT, (cells/mm^3^), median (IQR)	333 (130–521)	308 (110–500)	344 (136–521)	340 (132–535)	324 (141–512)	0.393
CD8+T at GRT, (cells/mm^3^), median (IQR)	725 (460–1085)	776 (511–1194)	807 (501–1137)	682 (429–1011)	697 (456–996)	<0.0001
Time between diagnosis and GRT, (weeks), median (IQR)	2.0 (0.8–5.0)	2.7 (1.2–6.4)	2.3 (1.0–7.1)	1.8 (0.7–4.4)	1.4 (0.6–3.4)	<0.0001
Origin, n (%)						
Italy	2,603 (78.2)	446 (80.5)	668 (80.9)	803 (80.1)	686 (72.6)	<0.0001
Europe	200 (6.0)	31 (5.6)	53 (6.4)	66 (6.6)	50 (5.3)	0.973
Asia	34 (1.0)	5 (0.9)	9 (1.1)	12 (1.2)	8 (0.8)	0.929
Africa	57 (1.7)	9 (1.6)	21 (2.5)	14 (1.4)	13 (1.4)	0.272
North America	13 (0.4)	2 (0.4)	5 (0.6)	5 (0.5)	1 (0.1)	0.368
South America	239 (7.2)	50 (9.0)	53 (6.4)	73 (7.3)	63 (6.7)	0.625
Unknown	181 (5.4)	10 (1.8)	17 (2.1)	30 (3.0)	124 (13.1)	<0.0001
Recent infections^b^, n (%)	210 (21.1)	51 (21.3)	69 (22.5)	58 (20.1)	32 (20.1)	0.618
Risk factor, n (%)						
Homosexual	1,654 (49.7)	268 (48.4)	404 (48.9)	538 (53.6)	444 (47.0)	0.599
Heterosexual	940 (28.2)	184 (33.2)	266 (32.2)	270 (26.9)	220 (23.3)	<0.0001
Bisexual	190 (5.7)	22 (4.0)	26 (3.1)	60 (6.0)	82 (8.7)	<0.0001
Drug user	194 (5.8)	58 (10.5)	47 (5.7)	57 (5.7)	32 (3.4)	<0.0001
Other/unknown	350 (10.5)	22 (4.0)	83 (10.0)	78 (7.8)	167 (17.7)	<0.0001
Number of sequences in TCs, n (%)	1,119 (33.6)	109 (19.7)	285 (34.5)	391 (39.0)	334 (35.3)	<0.0001

^a^By Kruskal-Wallis or Chi-squared test for trend, as appropriate.

^b^Information of chronic or recent infection was available for 994 individuals.

GRT: Genotypic resistance test; IQR: Interquartile-range; TC: Transmission Clusters.

Looking at the overall population, the majority of individuals were male (2,814, 84.6%), and Italian (2,603, 78.2%) with a median (Interquartile-range, IQR) age of 38 (31–46) years. Among the risk factors, homosexual and heterosexual contacts were the most prevalent infection routes (1,654 [49.8%] and 940 [28.2%], respectively). Median (IQR) plasma viral load (copies/mL) was 76,619 (21,216–258,222), median (IQR) CD4+T and CD8+T cell counts were 333 (130–521) and 725 (460–1,085) (cell/mm^3^), respectively (Table 1). Recent infections accounted for 21.1% (N = 210) of the individuals for whom information regarding the chronic or recent phase of infection was available. The majority of these individuals with recent infection were male (180, 85.7%) and Italian (178, 84.8%) with a median (Interquartile-range, IQR) age of 36 (30–43) years. Homosexual and heterosexual contacts were the most prevalent infection routes (144 [68.6%] and 45 [21.4%], respectively). Median (IQR) plasma viral load (copies/mL) was 54,288 (13,077–189,192), median (IQR) CD4+T and CD8+T cell counts were 535 (405–721) and 780 (582–1,237) (cell/mm^3^), respectively.

By looking at the four time spans, no statistically significant differences were observed with respect to demographic characteristics, with the exception of a slight increase in males, occurring in 2007–2009, and a slight decrease in Italians, in 2013–2016, and in heterosexuals, since 2010–2012 (Table 1). CD8+T cell count significantly decreased in 2010–2012 and remained stable in 2013–2016 (Table 1).

### Genetic distance evaluation over time

**Figure 1:**
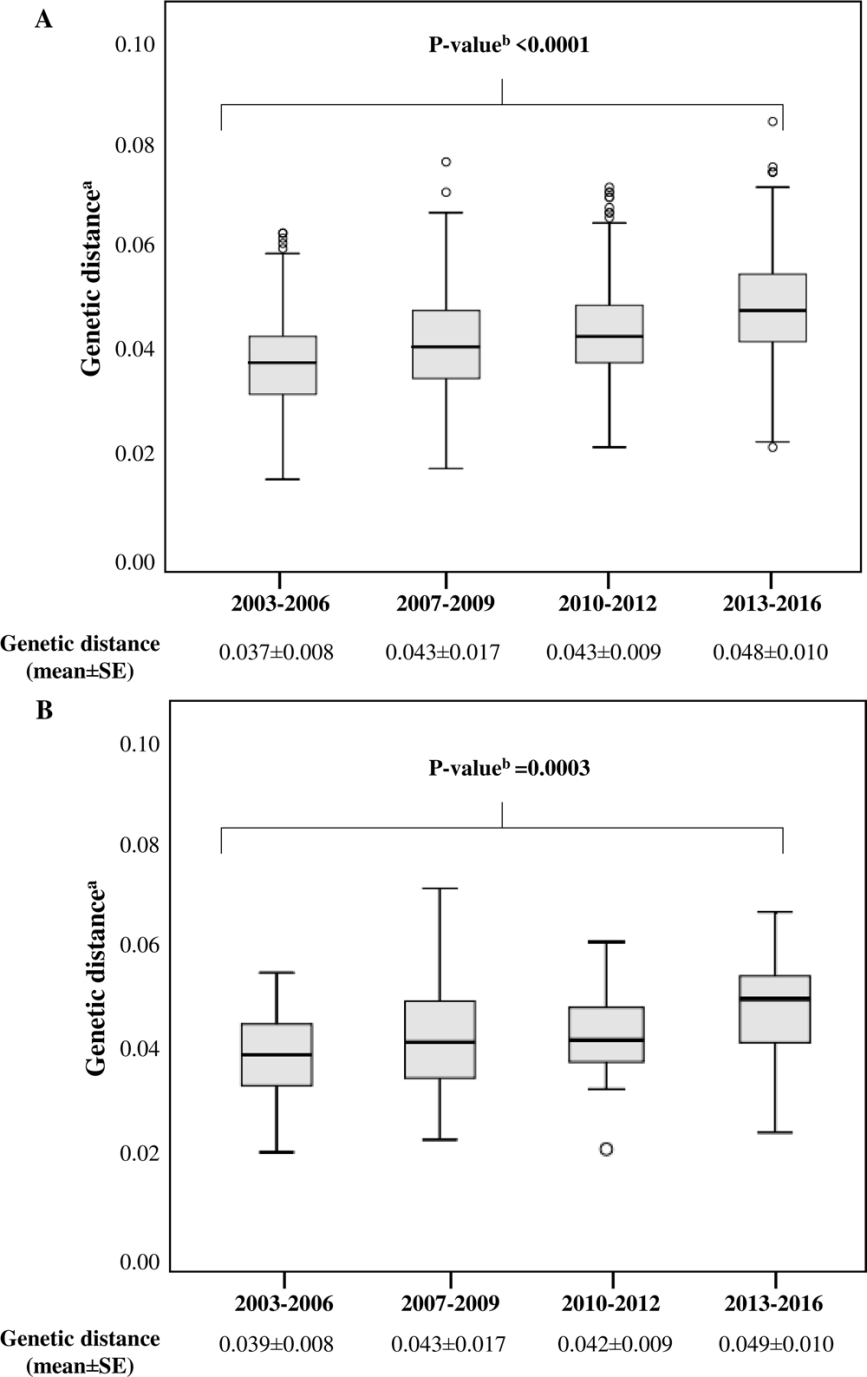
Genetic distance of 3,328 (**A**) and 210 (**B**) HIV-1 B subtype *pol* sequences, belonging to newly diagnosed and recently infected patients, respectively, divided into four time frames in accordance with year of diagnosis. ^a^Genetic distance was calculated by comparing sequences with HIV-1 consensus B using Tajima Nei model, MEGA 6. ^b^By Kruskal-Wallis test, corrected for Benjamini-Hochberg method. SE: standard error.

**Figure 2:**
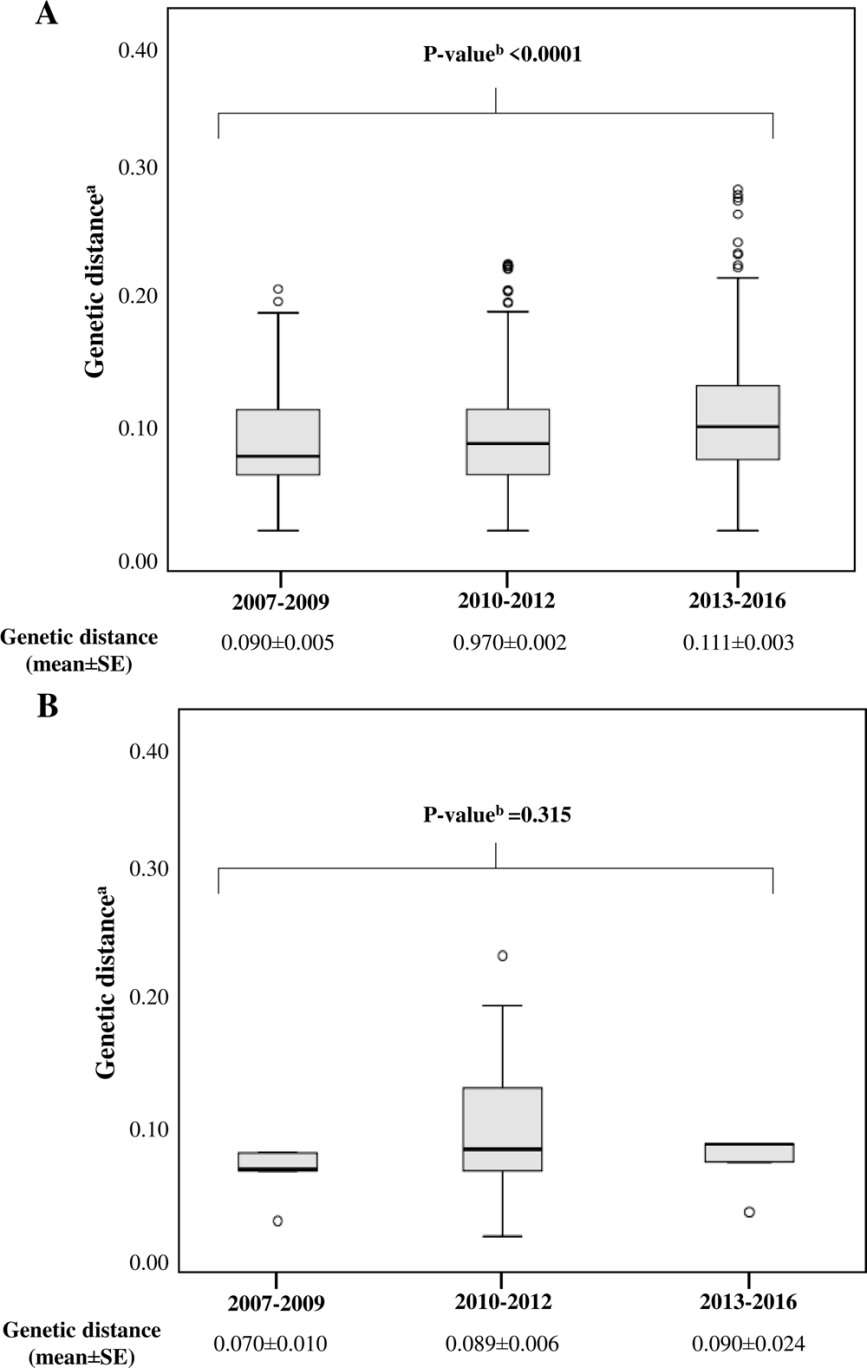
Genetic distance of 1,152 (**A**) and 66 (**B**) HIV-1 B subtype V3 sequences, belonging to newly diagnosed and recently infected patients, respectively, divided into three time frames. ^a^Genetic distance was calculated by comparing HIV-1 sequences with HIV-1 consensus B using Tajima Nei model, MEGA 6. ^b^By Kruskal-Wallis test, corrected for Benjamini-Hochberg method. SE: standard error.

In the overall population, the mean value of genetic distance (mean ± standard error, SE) compared with consensus B was 0.043 ± 0.012 for *pol* and 0.100 ± 0.001 for *V3*. A progressive and significant increase in genetic divergence from consensus B was observed over time for both *pol* and *V3* sequences (*pol*: from 0.037 ± 0.008 in 2003–2006 to 0.048 ± 0.010 in 2013–2016, P < 0.0001; *V3*: from 0.090 ± 0.005 in 2007–2009 to 0.111 ± 0.003 in 2013–2016, P < 0.0001) (Figs 1A and 2A). Comparable results were obtained considering only the recent infections with available 210 *pol* and 66 *V3* sequences, even if the statistical significance was obtained only for *pol* (*pol*: from 0.039 ± 0.008 in 2003–2006 to 0.049 ± 0.010 in 2013–2016, P = 0.0003; *V3*: from 0.070 ± 0.010 in 2007–2009 to 0.090 ± 0.024 in 2013–2016, P = 0.315) (Figs 1B and 2B).

Similar results were also obtained for dN and dS values (overall dN and dS, mean ± SE for *pol*: 0.019 ± 0.006 and 0.130 ± 0.043; overall dN and dS, mean ± SE for *V3*: 0.078 ± 0.001 and 0.129 ± 0.003). In particular, both dN and dS progressively increased over time in both *pol* and *V3* sequences (*pol*: from 0.017 ± 0.005 and 0.109 ± 0.029 in 2003–2006 to 0.021 ± 0.006 and 0.146 ± 0.037 in 2013–2016, P = 0.023 and 0.0001; *V3*: from 0.070 ± 0.004 and 0.117 ± 0.008 in 2007–2009 to 0.086 ± 0.002 and 0.139 ± 0.005 in 2013–2016, P = 0.0006 and 0.056) (Table 2 and Supplementary Material 1). Comparable results were again obtained considering only the recent infections (Table 2 and Supplementary Material 1).

**Table 2:** dN and dS parameters in accordance with the year of diagnosis in 3,328 and 210 HIV-1 B subtype *pol* sequences belonging to newly diagnosed and recently infected patients, respectively, divided into four time frames.

Year of diagnosis	*Pol* new diagnoses (N = 3,328)				*Pol* recent infections (N = 210)			
	dN	P-value	dS	P-value	dN	P-value	dS	P-value
2003–2006	0.017 ± 0.005	0.023	0.109 ± 0.029	0.0001	0.018 ± 0.005	0.023	0.018 ± 0.005	0.0001
2007–2009	0.018 ± 0.007		0.131 ± 0.062		0.019 ± 0.007		0.019 ± 0.007	
2010–2012	0.019 ± 0.006		0.128 ± 0.031		0.019 ± 0.005		0.019 ± 0.005	
2013–2016	0.021 ± 0.006		0.146 ± 0.037		0.021 ± 0.004		0.021 ± 0.004	

dN and dS parameters (espressed as mean ± SE) were calculated by comparing sequences with HIV-1 Consensus B by SNAP. Statistically significant differences were assessed by Kruskal-Wallis test, corrected for Benjamini-Hochberg method.

### Trend of amino acid variability over time

In order to measure the variation in *pol* and V3 amino acids over time, the first 369 amino acids of *pol* (99 PR amino acids and 270 reverse transcriptase [RT] amino acids) and the 35 amino acids defining the *V3* were analysed for the entropy value. In particular, *pol* amino acid sequences obtained in the period 2003–2006 were compared with those obtained in the period 2013–2016. The same analysis was performed for V3 amino acid sequences obtained in the period 2007–2009 and in the period 2013–2016.

Significant increases in entropy values were found at 18 positions (16 in *pol* and two in *V3*).

**Figure 3:**
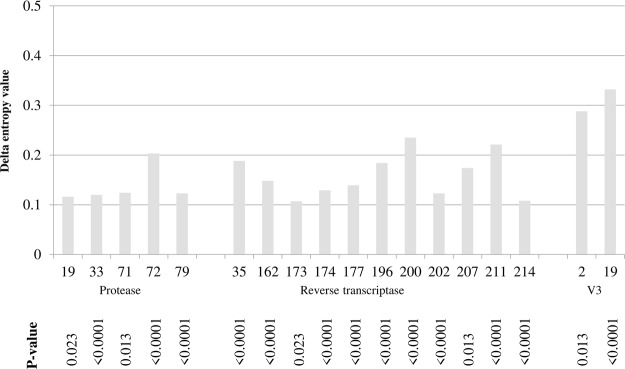
Amino acid positions characterized by an increased entropy value in the last time frame. *Pol* sequences obtained in the period 2003–2006 and those obtained in the period 2013–2016 were submitted for entropy analysis at HIV Los Alamos National Laboratory Entropy-Two tool (https://www.hiv.lanl.gov/content/sequence/ENTROPY/entropy.html) in order to obtain delta entropy values. The same analysis was performed by comparing V3 sequences obtained in the period 2007–2009 and in the period 2013–2016. Only delta values ≤0.10 and >0.10 followed by a P ≤ 0.05 after Benjamini-Hockberg correction were considered statistically significant.

In particular, by comparing *pol* amino acid sequences, entropy increased at five amino acid positions in protease (PR: 19, 33, 71, 72, 79), and at 11 positions in RT (35, 162, 173, 174, 177, 196, 200, 202, 207, 211, 214) (Fig. 3). Interestingly, CTL escape variants occur at seven of these positions, such as the PR position 71 (escape variant: A71V), and the RT positions 35 (escape variant: V35I), 162 (escape variant: S162ACQR), 177 (escape variant: D177E), 202 (escape variant: I202V), 207 (escape variant: Q207GE) and 211 (escape variant: R211K). By comparing *V3* amino acid sequences, entropy increased at positions 2 and 19 (Fig. 3).

Entropy significantly decreased only at RT amino acid position 11 (delta entropy value: −0.122, P = 0.0001).

### Trend of CTL escapes over time

By analysing the prevalence of CTL escape mutations in *pol*, we found that 8.7% of sequences (N = 288) did not contain escape mutations, 19.0% (N = 632) contained one escape, 26.2% (N = 872) contained two escapes, 23.0% (N = 767) contained three escapes, 13.9% (N = 462) contained four escapes, and 9.2% (N = 307) contained ≥5 escapes. Among recent infections, 8.6% of sequences (N = 18) did not contain escape mutations, 21.4% (N = 45) contained one escape, 30.0% (N = 63) contained two escapes, and 40.0% (N = 84) contained ≥3 escapes. Looking at the CTL escape prevalence over time, *pol* sequences with ≥3 CTL escapes rose from 36.1% in 2003–2006 to 54.0% in 2013–2016 (P < 0.0001). In particular, *pol* sequences with 3, 4 and ≥5 CTL escapes increased from 18.9%, 11.4%, and 5.8% in 2003–2006 to 25.3%, 16.5% and 12.2% in 2013–2016, P = 0.008, 0.004, and <0.0001, respectively (Table 3). This increase was mainly due to PR escape mutation A71V, and RT escape mutations S162ACQR, D177E, I202V, and R211K (Supplementary Material 2). Limiting the analysis to recent infections, *pol* sequences with ≥3 CTL escapes were more frequently found in 2013–2016 compared with 2003–2006 (56.2% vs. 31.4%), even if this increase did not reach statistical significance (P = 0.139) (Table 4).

**Table 3:** Prevalence of HIV-1 B subtype *pol* sequences with 0, 1, 2, 3, 4 and ≥5 CTL escape mutations, belonging to 3,328 newly diagnosed patients, divided into four time frames in accordance with year of diagnosis.

Number of CTL escapes	Overall HIV-1 B subtype new diagnoses (N = 3,328)				P-value
	2003–2006, N = 554, n (%)	2007–2009, N = 826, n (%)	2010–2012, N = 1,003, n (%)	2013–2016, N = 945, n (%)	
0, N = 288	58 (10.5)	79 (9.6)	78 (7.8)	73 (7.7)	0.036
1, N = 632	130 (23.4)	168 (20.3)	188 (18.7)	146 (15.4)	<0.0001
2, N = 872	166 (30.0)	213 (25.8)	277 (27.6)	216 (22.9)	0.012
3, N = 767	105 (18.9)	186 (22.5)	237 (23.6)	239 (25.3)	0.008
4, N = 462	63 (11.4)	105 (12.7)	138 (13.8)	156 (16.5)	0.004
≥5, N = 307	32 (5.8)	75 (9.1)	85 (8.5)	115 (12.2)	<0.0001

Chi-squared test for trend, corrected for Benjamini-Hochberg method, were used to estimate significant changes over the four time periods.

**Table 4:** Prevalence of HIV-1 B subtype *pol* sequences with 0, 1, 2, and ≥3 CTL escape mutations, belonging to 210 recently infected patients, divided into four time frames in accordance with year of diagnosis.

Number of CTL escapes	Overall HIV-1 B subtype recent infections (N = 210)				P-value
	2003–2006, N = 51, n (%)	2007–2009, N = 69, n (%)	2010–2012, N = 58, n (%)	2013–2016, N = 32, n (%)	
0, N = 18	3 (5.9)	7 (10.1)	5 (8.6)	3 (9.4)	0.640
1, N = 45	14 (27.4)	14 (20.4)	13 (22.4)	4 (12.5)	0.171
2, N = 63	18 (35.3)	17 (24.6)	21 (36.2)	7 (21.9)	0.521
≥3, N = 84	16 (31.4)	31 (44.9)	19 (32.8)	18 (56.2)	0.139

Chi-squared test for trend, corrected for Benjamini-Hochberg method, were used to estimate significant changes over the four time periods.

By analysing significant patterns of pairwise correlations among *pol* CTL escape mutations, a positive significant correlation was found between the CTL escapes at position 162 (S162ACQR) and the escape at position 177 (D177E) (number of sequences showing the correlated pair = 169; covariation frequency: 35.1%; phi = 0.11, P < 0.0001).

By analysing V3 loop, only 8 sequences in 1,152 (0.7%) contained CTL escapes. All these sequences belonged to the time frames 2010–2012 and 2013–2016 (2 [0.3%] and 6 [1.7%], respectively).

### Role of epidemiological confounders in influencing HIV-1 B subtype variability and CTL escapes

We next sought to investigate whether changes in risk factors, in patients’ origin, and in sequences involved in transmission clusters (TCs) across time might interfere with HIV-1 B subtype increased variability.

Firstly, we investigated whether TCs frequency correlates with HIV-1 increased variability by comparing the subgroup of 1,119 *pol* sequences involved in TCs with the subgroup of 2,209 *pol* sequences out of TCs. The progressive and significant increase in genetic divergence from consensus B over time was confirmed both for sequences in TCs and for sequences out of TCs (TCs: from 0.038 ± 0.001 in 2003–2006 to 0.047 ± 0.00001 in 2013–2016, P < 0.0001; out of TCs: from 0.037 ± 0.00001 in 2003–2006 to 0.048 ± 0.00001 in 2013–2016, P < 0.0001). Similar results were also obtained for dN and dS values (TCs: from 0.016 ± 0.0004 and 0.113 ± 0.003 in 2003–2006 to 0.020 ± 0.0003 and 0.143 ± 0.002 in 2013–2016, P < 0.0001 and <0.0001; out of TCs: from 0.017 ± 0.0003 and 0.107 ± 0.001 in 2003–2006 to 0.021 ± 0.0002 and 0.147 ± 0.002 in 2013–2016, P < 0.0001 and <0.0001).

Looking at the CTL escape prevalence over time, *pol* sequences with 3, 4 and ≥5 CTL escapes increased both in TCs and out of TCs, although not always significantly (in TCs: from 24.0%, 8.3%, and 5.5% in 2003–2006 to 28.1%, 14.7% and 14.4% in 2013–2016, P = 0.209; P = 0.060; P = 0.012; out TCs: from 17.5%, 12.1%, and 5.8% in 2003–2006 to 23.7%, 17.5% and 11.0% in 2013–2016, P = 0.075; P = 0.020; P = 0.030).

In line with these results, the presence of TCs did not significantly affect either the number of circulating CTL escapes (Pearson’s phi = 0.015; P = 0.118) or the spread of single CTL escape (RT S162ACQR: phi = −0.25; P = 0.152; RT D177E: phi = 0.008; P = 0.641; RT I202V: phi = −0.051; P = 0.184; RT R211K; phi = −0.21; P = 0.221), with the exception of PR A71V (phi = 0.105; P < 0.0001).

Overall results suggest that the HIV-1 B subtype increased variability and the spread of CTL escapes are not predominantly correlated with expanding clades over time.

We next investigated whether variation in the proportion of risk factors, and patients’ origin across time might influence the HIV-1 B subtype increased variability. To do so, we restricted the analysis to a more homogenous population, composed of *pol* (N = 1,308) and *V3* (N = 449) sequences belonging to MSM of Italian origin. The results confirmed the progressive and significant increase in genetic divergence from consensus B over time for *pol*, and like a trend for *V3* (*pol*: from 0.038 ± 0.001 in 2003–2006 to 0.047 ± 0.001 in 2013–2016, P < 0.0001; *V3*: from 0.085 ± 0.007 in 2007–2009 to 0.098 ± 0.005 in 2013–2016, P = 0.348). Similar results were also obtained for dN and dS values (*pol*: from 0.017 ± 0.0004 and 0.110 ± 0.002 in 2003–2006 to 0.020 ± 0.0003 and 0.143 ± 0.002 in 2013–2016, P < 0.0001 and <0.0001; *V3*: from 0.072 ± 0.007 and 0.093 ± 0.012 in 2007–2009 to 0.077 ± 0.004 and 0.126 ± 0.010 in 2013–2016, P = 0.635 and 0.072). Looking at the CTL escape prevalence, *pol* sequences with 3, 4 and ≥5 CTL escapes were more frequently found in 2013–2016 compared with 2003–2006, even if this increase was not always significant (3 CTL escapes: from 20.8% in 2003–2006 to 26.6% in 2013–2016, P = 0.394; 4 CTL escapes: from 11.6% to 14.6%, P = 0.224; ≥5 CTL escapes: from 5.8% to 14%, P = 0.018). Again, these results suggest that changes in risk factors and patients’ origin are not predominantly responsible for HIV-1 B subtype variability.

### Impact of CTL escapes and year of diagnosis on HIV-1 *pol* evolution

A subgroup of 153 individuals with two *pol* sequences available before therapy start (time-window between sequences within 70 weeks) was analysed to define if the evolutionary rate of HIV-1 per site per year may be affected by the time of diagnosis and number of CTL escapes. The evolutionary rate of HIV-1 per site per year in the 153 pairs of sequences was 3.9 × 10^−3^ (3.3 × 10^−3^–4.5 × 10^−3^).

Among these pairs, 73 carried ≥3 CTL escapes (time-window between sequences, median weeks [IQR] 59 [44–83]), while 80 carried ≤2 CTL escapes (time-window between sequences, median weeks [IQR] 62 [41–77]). Stratifying the pairs based on year of diagnosis, 28 pairs carrying ≥3 CTL escapes belonged to patients diagnosed after 2009 (time-window between sequences, median weeks [IQR] 58 [45–90]), while 57 carrying ≤2 CTL escapes belonged to patients diagnosed before 2009 (time-window between sequences, median weeks [IQR] 66 [51–78]).

Rates of HIV-1 intra-host evolution (95% confidence interval, CI) were consistently lower in pairs containing ≥3 CTL escapes than in those containing ≤2 CTL escapes (3.0 × 10^−3^ [2.9 × 10^−3^−3.1 × 10^−3^] vs. 4.3 × 10^−3^ [4.0 × 10^−3^–5.0 × 10^−3^], P [likelihood-ratio test, LRT] < 0.0001[21.09]). Consistent with these data, the selective pressure was lower in *pol* containing ≥3 CTL escapes than in *pol* containing ≤2 CTL escapes (dN/dS, 95% CI: 0.28 [0.24–0.34] vs. 0.25 [0.22–0.29]).

Stratifying the pairs based on year of diagnosis, the rates of HIV-1 intra-host evolution (95% CI) were significantly lower in pairs containing ≥3 CTL escapes and belonging to patients diagnosed after 2009 than in those with ≤2 CTL escapes and belonging to patients diagnosed before 2009 (3.2 × 10^−3^ [3.0 × 10^−3^–4.0 × 10^−3^] vs. 4.5 × 10^−3^ [4.0 × 10^−3^–5.0 × 10^−3^], P [LRT] < 0.001[12.76]). Again the selective pressure was confirmed to be lower in sequences circulating after 2009 and with ≥3 CTL escapes than in those circulating before 2009 and with ≤2 CTL escapes (dN/dS, 95% CI: 0.21 [0.15–0.27] vs. 0.27 [0.23–0.32]).

These findings might suggest a better HIV-1 intra-host adaptation for most recent viruses driving a higher number of CTL escapes.

### Correlation between *pol* CTL escape mutants and viro-immunological parameters

By analysing a subgroup of 781 individuals characterized by recent infection or plasma viral load >10,000 copies/ml and CD4+T cells >500 cells/mm^3^, no significant correlations were found between CTL escapes and plasma viral load or CD4+T cell counts.

On the other hand, CD4/CD8 ratio significantly decreased in relation to the increased number of CTL escapes (from 1.64 ± 0.68 in patients infected by a virus with 0 CTL escapes to 0.69 ± 0.05 in patients infected by a virus with ≥5 CTL escapes, ρ = −0.100, P = 0.027) (Table 5). The negative correlations between the number of CTL escapes in *pol* and lower CD4/CD8 ratio were confirmed also when only the recent infections were analysed (1.02 ± 0.18 for 0 CTL escapes vs. 0.74 ± 0.06 for ≥3 CTL escapes, ρ = −0.15, P = 0.067) (Table 6).

**Table 5:** Correlation between CTL escape mutations and viro-immunological parameters belonging to 781 individuals characterized by recent infection or by plasma viral load >10,000 copies/ml and CD4+T cells >500 copies/ml.

Number of CTL escapes	Plasma viral load, copies/ml, median (IQR)	P-value	CD4+T cells, cells/mm^3^, median (IQR)	P-value	CD4/CD8 Ratio, mean ± SE	P-value
0, N = 75	46,955 (26,054–139,500)	0.994^*^ 0.667^§^	690 (536–816)	0.913^*^ 0.912^§^	1.64 ± 0.68	0.034^*^ 0.027^§^
1, N = 147	60,000 (21,670–223,238)		621 (535–764)		1.02 ± 0.15	
2, N = 224	56,160 (23,296–170,885)		628 (542–764)		0.82 ± 0.05	
3, N = 175	45,973 (162,295-125,728)		629 (539–777)		0.82 ± 0.05	
4, N = 90	69,285 (24,275–262,493)		664 (560–800)		0.79 ± 0.07	
≥5, N = 70	53,904 (18,213–205,785)		603 (549–695)		0.69 ± 0.05	

^*^Statistically significant differences were assessed by Kruskal-Wallis test, corrected for Benjamini-Hochberg method. ^§^Statistically significant differences were assessed by Spearman correlation. IQR: Interquartile-range; SE: standard error.

**Table 6:** Correlation between CTL escape mutations and viro-immunological parameters belonging to 210 recently infected patients.

Number of CTL escapes	Plasma viral load, copies/ml, median (IQR)	P-value	CD4+T cells, cells/mm^3^, median (IQR)	P-value	CD4/CD8 Ratio, mean ± SE	P-value
0, N = 18	46,364 (36,589–93,896)	0.820^*^ 0.442^§^	477 (395–720)	0.970^*^ 0.708^§^	1.02 ± 0.18	0.309^*^ 0.067^§^
1, N = 45	54,740 (17,168–371,500)		574 (430–728)		1.33 ± 0.41	
2, N = 63	61,690 (13,125–184,514)		538 (429–697)		0.84 ± 0.11	
≥3, N = 84	43,410 (9,767–231,416)		539 (407–777)		0.74 ± 0.06	

^*^Statistically significant differences were assessed by Kruskal-Wallis test, corrected for Benjamini-Hochberg method. ^§^Statistically significant differences were assessed by Spearman correlation. IQR: Interquartile-range; SE: standard error.

## Discussion

Our analysis based on HIV-1 B subtype *pol* (N = 3,328) and *V3* (N = 1,152) sequences collected from a large number of HIV-1 newly diagnosed individuals in Italy in the time frame 2003–2016 indicates a progressive and significant increase in genetic and amino acid distance over time (with 18 amino acid positions characterized by entropy increase) for the most disseminated HIV-1 subtype in the world. This finding is also confirmed when only the subgroup of recently infected individuals is taken into account, confirming the higher variability of the new circulating viral strains belonging to HIV-1 B subtype compared with the older.

Despite the short time since its emergence (dated in 1944), HIV-1 B has generated successful epidemics in many countries in five continents, and the spread continues^21^. Due to the global extension of the HIV-1 B epidemic^22–24^, and the high evolutionary rates characterizing HIV^25^, a progressive diversity over time for this subtype has been reported both in North America^20^ and, even if limited to protease and integrase, in Europe^26,27^. A recent paper comparing the historic (1979–1989) versus modern (post-2000) HIV-1 subtype B cohorts in North America showed that in the past 30 years HIV-1 diversity increased approximately twofold, and the average background frequencies of CTL escape mutations also doubled^20^.

In our dataset, seven out of the 18 positions characterized by entropy increase take part in CTL escape variants (PR 71; RT 35, 162, 177, 202, 207, 211).

Of note, prevalence of CTL escapes significantly increased in the last time frame at five of these seven positions (PR: A71V; RT: S162ACQR, D177E, I202V, and R211K). Whereas entropy at positions 35 and 207 increased, V35I and Q207GE escapes did not change their prevalence concomitantly (V35I and Q207G/E prevalence: 18.8% and 19.1% in 2003–2006 vs. 16.8% and 19.4% in 2013–2016, P = 0.672 and P = 0.119, respectively). The reason for the entropy increase at these 2 positions may thus reside in other amino acid mutations not involved in CTL escapes, such as V35LTM and Q207HNADRK, whose prevalence increased from 7.9% and 8.1% in 2003–2006 to 13.4% and 10.7% in 2013–2016, respectively (P = 0.034 and P = 0.048). The role of these amino acid mutations is not clearly demonstrated, and thus further studies might be necessary to define their impact on HIV-1 B evolution and pathogenesis.

Notwithstanding the increase in sequence diversity observed over time in our dataset, we have to consider that HIV variation and immune escape process are also limited by structural and functional constraints^28,29^. In particular, the virus would have fewer viable options to generate escape variants in conserved protein regions because of the fitness cost required^30^. However, HIV overcomes this problem thanks to specific mechanisms that compensate for the fitness costs. For example, studies on *gag* polyprotein demonstrated the selection of compensatory mutations, which can restore the viral fitness. These mutations can occur both within^31^ and outside the epitope, but nearby in the three-dimensional protein structure^32,33^.

In this respect, the increased prevalence of some amino acid variants in *pol* and their correlation in mutational pathways (such as for S162ACQR and D177E) may suggest a compensatory role played by these mutations, which can therefore explain their frequent occurrence, rather than by chance in the circulating viral population. Its effect may positively influence the fixation of a specific CTL escape at population level^34^. In line with this, the prevalence of sequences with ≥3CTL escapes progressively increased in both overall population and recent infections, reaching 54.0% and 56.2% in 2013–2016, respectively. This may represent an evolutionary advantage with consequences for disease progression, infectivity, transmissibility, and response to antiviral treatments^35^.

Moreover, the ability of HIV-1 immune escape mutations to accumulate rapidly could undermine host antiviral immunity^36–38^, and thus in turn have immunological consequences at population level. In this respect, our study found that the higher number of CTL escapes in *pol* frequently correlated with lower CD4/CD8 ratio (delta CD4/CD8 ratio = −0.95) at HIV-1 diagnosis, suggesting an increased pathogenic potential of these viral strains. However, these data need to be interpreted with caution, especially because of the lack of HLA information.

The impossibility to match CTL escape variants with HLA types of the host also represents a limitation for the estimation of the CTL escape prevalence. It is known that CTL escape prevalence may differ between HLA mismatched and HLA matched people^39,40^, and that the fixation of some HIV-1 polymorphisms at population level strongly depends on population characteristics^41^. To partially overcome the lack of HLA information, we have focused attention on those CTL escapes that significantly increased over time, such as the PR A71V and the RT D177E (related to HLA-B*1503), the RT S162ACQR (related to HLA-A*1101 –A*8601, -A*0301, -B*38 and B*0702), I202V (related to HLA-B4001), and R211K (related to HLA-A*2501). By matching these data with the prevalence of HLA in Italian population, we found that all the mentioned HLA are well represented in Italy. In particular, the HLA-A*1101, -A*2501 and -A*0301 represent 16% of the overall HLA-A locus, while the HLA- B*5101, -B*0702, -B*4001, and -B*03 16% of the overall HLA-B locus^42^. Thus, the spread of the previously mentioned CTL escape polymorphisms at the population level in Italy may be explained by the relative diffusion of matched HLA in the general Italian population.

However, the persistence of CTL escapes is not always dependent on matched HLA. Specific escape mutations persist upon transmission to HLA unmatched hosts, perhaps owing to a lack of fitness costs^43,44^. A recent mathematical model shows that the median time to escape in HLA matched individuals across the 26 epitopes considered is 8 years, and the reversion rates is a little slower (median time to reversion: 6.5 years) with no evidence of reversion in 56% of CTL epitopes, suggesting that CTL escapes are able to spread rapidly at population level independently of HLA^45^.

The role of CTL escapes in the process of HIV adaptation to the host was also confirmed by the estimation of the intra-host evolution. In particular, by analysing a subgroup of individuals with two *pol* sequences available, we found that the rate of intra-host evolution and the consequent selective pressure were significantly lower for most recent HIV-1 diagnoses carrying ≥3 CTL escapes than older diagnoses carrying fewer CTL escapes (3.2 × 10^−3^ vs. 4.5 × 10^−3^). In the context of the already known slow HIV-1 *pol* evolution^46^, the slower intra-host evolution found for most recent circulating viruses driving a higher number of CTL escapes may suggest that HIV-1 had improved its adaptation to the host in recent years compared with the past, even if the adaptation process is still occurring.

Besides CTL escapes, further attention should be given to neutralizing epitope escapes, of which the *V3* loop is rich. In our study, we found that while in *pol* entropy values increased also within CTL epitopes (such as PR position 71 and the RT positions 35, 162, 177, 202, 207 and 211), in *V3* higher mutation rates occurred almost exclusively within previously described neutralizing epitopes^47^, and at residue 19 already associated with R5 tropism^48^. We are conscious that our analysis is limited to a very short region of *env*, and that further studies taking into account a longer *env* fragment encompassing at least all the variable loops and conserved regions of gp120 may be necessary. Similar studies will be of extreme interest in defining the tracing of amino acid modifications in HIV-1 domains involved in vaccine strategies and co-receptor affinity.

Looking at potential epidemiological confounders able to influence HIV-1 B subtype variability and CTL escapes over time, we found that neither risk factor nor patients’ origin, nor TCs strictly (and exclusively) impact HIV-1 B subtype variability over time. Doing these sub-studies yielded results mainly consistent with the overall analysis, suggesting that HIV-1 B subtype varied over time, regardless of these confounders.

Nevertheless, we cannot definitely exclude that the relevant epidemiological changes occurring in Italy in recent years, such as the migratory waves from low-middle income areas^49^, or the time-dependent influx of sequences from different risk groups might contribute (even if not predominantly) to HIV-1 B subtype variation in this country. For example, in our dataset individuals of “unknown” origin and “other or unknown” risk factors increased from 1.8% and 4.0% in 2003–2006 to 13.1% and 17.7% in 2013–2016, while Italians and heterosexuals decreased from 80.5% and 33.2% in 2003–2006 to 72.6% and 23.3% in 2013–2016. This geographic and epidemiological admixture combined with the documented high rate of post-immigration HIV-1 acquisition^50^ might also favour the diffusion of new HLAs at population level and the selection of new CTL escape mutations.

Finally, as the article is mainly focused on *pol*, the introduction of antiretroviral therapies merits attention (RT inhibitors were introduced in the late 1980s and PR inhibitors in the mid 1990s). Indeed, two out of the 18 positions characterized by entropy increase, the PR 33 and 71, are involved in drug resistance. The observed entropy increase was mainly due to the mutations L33F/I and A71VTIL, whose prevalence increased in the last two time frames (L33FI: 0.4% in 2003–2006 vs.1.2% in 2007–2009 vs. 3.9% in 2010–2012 vs. 3.0% in 2013–2016, P < 0.0001; A71VTIL: 15.0% in 2003–2006 vs. 13.7% in 2007–2009 vs. 18.5% in 2010–2012 vs. 21.2% in 2013–2016, P < 0.0001). Both L33F and A71VTIL are PI-selected accessory mutations, associated with a minimal or null reduced susceptibility to PIs^51,52^. Even if some of these mutations such as L33I and A71VT may also appear as natural polymorphisms or CTL escapes (such as A71V), we cannot exclude that their increased prevalence in the last two time frames may be an indicator of viral adaptation to PI drug exposure.

In conclusion, this study, based on HIV-1 B subtype sequences diagnosed in Italy, provides additional evidence that HIV-1 is still adapting to host, and that this adaptation may include CTL escape mutations. These findings can help in making accurate maps of escape pathways and in defining optimal induction CTLs strategies that may be effective in controlling HIV-1 replication, which may have implications for immunotherapeutic interventions. Moreover, this paper poses the bases for further studies involving viral evolution and viral adaptation focused on rapidly expanding epidemics such as those currently occurring in Africa and Asia, mainly driven by recombinant forms and non-B pure forms.

## Materials and Methods

### Study population

This study was conducted on 3,328 *pol* sequences (containing the full-length PR and the first 270 RT codons; nucleotides length: 1,107) and 1,152 *V3* sequences (nucleotides length: 105; amino acid length: 35) obtained from HIV-1 B subtype newly diagnosed infected individuals (one sequence per patient) in Italy. All patients were naïve to cART and 210 of them were HIV-1 recently infected. *Pol* and *V3* sequences were performed from plasma samples for routine clinical purposes from 2003 to 2016 and from 2007 to 2016, respectively, immediately after the diagnosis, and then collected in an anonymous database^53,54^. HIV-1 B subtype for *pol* and *V3* sequences was firstly determined by Stanford DB (https://hivdb.stanford.edu/) and geno2pheno algorithm (https://coreceptor.geno2pheno.org/), respectively, and further confirmed by phylogenetic analysis based on *pol*, as described previously^55^.

Recent infections were determined by: (i) clinical/laboratory signs of primary HIV infection (HIV-1 plasma viral load levels >10,000 copies/mL and negative or indeterminate HIV-1 antibody test); (ii) a documented negative HIV-1 test performed within the 6 months prior to the HIV-1 diagnosis; and (iii) an antibody avidity index ≤0.80 (test performed only in clinically AIDS free individuals)^56,57^.

### Ethic statement

This study was conducted on data collected for clinical purposes. All data used in the study were previously anonymized, according to the requirements set by Italian Data Protection Code (leg. decree 196/2003) and by the General authorizations issued by the Data Protection Authority. This study was approved by the ethics committee of Tor Vergata Hospital (Ethics Approval No. 238/16, 14 December 2016) and L. Spallanzani National Institute for Infectious Diseases, IRCCS (Ethics Approval No. 51, 18 December 2003).

### Genetic distance and non-synonymous and synonymous substitution rate estimation

Genetic distance between each *pol* and *V3* sequence and the HIV-1 subtype B consensus sequence was evaluated using MEGA 6 program based on the Tajima Nei model^46,58^.

The HIV-1 subtype B consensus sequence used for this analysis was published in 2002 by Los Alamos HIV Database. This consensus can be retrieved at https://www.hiv.lanl.gov/content/sequence/NEWALIGN/align.html site. The consensus sequence was calculated according to the default values on the Consensus Maker tools. Details can be retrieved at https://www.hiv.lanl.gov/content/sequence/NEWALIGN/align.html#consensus.

Mean number of synonymous (dS) and nonsynonymous (dN) substitutions per site were calculated by the method of Nei and Gojobori^59^, through the automatic tool SNAP (https://www.hiv.lanl.gov/content/sequence/SNAP/SNAP.html).

### CD8+ cytotoxic T-lymphocyte (CTL) escape mutations

Prevalence of CTL escape mutations in each *pol* and *V3* sequence was evaluated considering all variants in the Los Alamos Immunology Database list defined as: documented escape, or non-susceptible form, or calculated escape, or inferred escape, or diminished response escape (https://www.hiv.lanl.gov/content/immunology/variants/ctl_variant.html, Data last updated at 2017-03-09, Supplementary Material 3).

To identify potential patterns of pairwise correlations between specific CTL escape mutations, a binomial correlation coefficient (phi) and its statistical significance for each pair of mutations were calculated. Average linkage hierarchical agglomerative cluster analysis was performed to investigate potential evolutionary pathways among CTL escape mutations^60^.

### Shannon Entropy calculation

In order to evaluate the variability of each amino acid site in *pol* over time, the first 369 amino acids of *pol* (99 PR amino acids and 270 RT amino acids) were analysed for the entropy value. In particular, amino acid sequences obtained in the period 2003–2006 and those obtained in the period 2013–2016 were submitted to HIV Los Alamos National Laboratory (LANL) Entropy-Two tool (https://www.hiv.lanl.gov/content/sequence/ENTROPY/entropy.html)^61^. The same analysis was performed by comparing entropy values at the 35 amino acid positions of *V3* sequences obtained in the period 2007–2009 and in the period 2013–2016. Only delta values ≤0.10 and >0.10 followed by a P ≤ 0.05 after Benjamini-Hochberg correction^62^ were considered statistically significant.

### Statistical analysis

In order to estimate differences in demographic, virological and immunological parameters, genetic distance and CTL escape prevalence over time, *pol* sequences were divided into four time spans in accordance with the year of diagnosis (time spans: 2003–2006, 2007–2009, 2010–2012, 2013–2016). For the same purposes, *V3* sequences, available from 2007, were divided into three time spans (time spans: 2007–2009, 2010–2012, 2013–2016).

CTL escapes were divided into 6 pre-defined categories (0, 1, 2, 3, 4, and ≥5 CTL escapes) for the overall population and into 4 pre-defined categories (0, 1, 2, and ≥3 CTL escapes) for recently infected individuals.

Genetic-distance, dN and dS values, and number of CTL escapes were also evaluated in 1) the subgroup of 1,308 *pol* and 449 *V3* sequences belonging to MSM of Italian origin, 2) the subgroup of 1,119 *pol* sequences involved in TCs compared with the subgroup of 2,209 *pol* sequences out of TCs for the purpose of investigating whether risk factors, patients’ origin, and sequences in TCs across time might interfere with obtained results. Potential TCs (defined as clusters of ≥3 *pol* sequences) were identified by HIV-TRACE using a Tamura-Nei model, and a genetic distance cut-off ≤0.015^63^.

Kruskal-Wallis and Chi-squared test for trend were used to estimate significant changes over time periods, while Pearson’s Chi-squared test and Mann Whitney test were used to estimate significant changes between two different time periods.

Spearman correlation and Kruskal-Wallis test were also used to estimate significant correlations between viro-immunological parameters and number of CTL escapes. In order to ensure, wherever possible, exclusion of individuals with a late diagnosed chronic infection, these analyses were restricted to a subgroup of 781 individuals including the documented recent infections (N = 210) and individuals characterized by plasma viral load >10,000 copies/ml and CD4+T cells >500 cells/mm^3^ (N = 571). Viral load and CD4+T cells cut-offs were chosen in accordance with Pantazis N. *et al*. Lancet HIV 2014^64^.

Again, only P ≤ 0.05 after Benjamini-Hochberg correction^62^ were considered statistically significant.

All the analyses were performed using the R open source statistical environment (version 3.3.1) and SPSS (version 23) for Windows (SPSS Inc., Chicago, Illinois).

### Evolutionary rate estimation

To determine if year of diagnosis and number of CTL escapes may affect the intra-patient evolutionary rate of HIV-1, a subgroup of 153 patients with two *pol* sequences available before therapy initiation (time-window between sequences within 70 weeks) were analyzed by using HyPhy *pol* evolution package^65^. All sequences from each patient were arranged chronologically and the probability of evolving from the oldest to the newest sequence was estimated using maximum likelihood method. In particular, the *pol* sequences of individuals diagnosed after 2009 and with ≥3 CTL escapes were compared with *pol* sequences of individuals diagnosed before 2009 and with ≤2 CTL escapes. Branch lengths between the two sequences were assigned based on the difference between sampling dates. For each individual, we determined the nucleotide substitution rate by using a General Time Reversible (6 substitution rates, empirical frequencies) model, assuming strict molecular clock. Significance was assessed using the likelihood ratio test (LRT) with N-2 degrees of freedom. The evolutionary rate per site per year within host were tested for equality using the likelihood-ratio test (LRT)^66^. Sequence data are available in the Supplementary Dataset 1.

## Electronic supplementary material

Supplementary Information

Supplementary Dataset 1
