# Supplementary material for: Genetic divergence of HIV-1 B subtype in Italy over the years 2003–2016 and impact on CTL escape prevalence
Source: Sci Rep. 2018 Oct 24;8:15739. doi: 10.1038/s41598-018-34058-7 (PMC6200748; doi:10.1038/s41598-018-34058-7)
Supplement: Supplementary file 1 — Supplementary Information [file 41598_2018_34058_MOESM1_ESM.pdf]

Genetic divergence of HIV-1 B subtype in Italy over the years 2003-2016 and impact on CTL escape prevalence

Claudia Alteri[1*+], Lavinia Fabeni[1+], Rossana Scutari[1], Giulia Berno[2], Domenico Di Carlo[3], Caterina Gori[2], Ada Bertoli[1], Alessandra Vergori[2], Ilaria Mastrorosa[2], Rita Bellagamba[2], Cristina Mussini[4], Manuela Colafigli[5], Francesco Montella[6], Alfredo Pennica[7], Claudio Maria Mastroianni[8], Enrico Girardi[2], Massimo Andreoni[9], Andrea Antinori[2], Valentina Svicher[1], Francesca Ceccherini-Silberstein[1], Carlo Federico Perno[2-10], Maria Mercedes Santoro[1]

[1]Department of Experimental Medicine and Surgery, University of Rome “Tor Vergata”, Rome, 00133, Italy
[2]National Institute for Infectious Diseases L. Spallanzani, IRCCS, Rome, 00161, Italy
[3]Department of Biomedical and Clinical Sciences "L. Sacco", University of Milan, Pediatric Clinical Research Center “Romeo and Enrica Invernizzi”, Milan, 20133, Italy
[4]University Hospital of Modena, Modena, 41121, Italy
[5]San Gallicano Hospital, IFO-IRCCS, Rome, 00144, Italy
[6]San Giovanni Hospital, Rome, 00184, Italy
[7]Sant’Andrea Hospital, Rome, 00189, Italy
[8]University of Rome “La Sapienza”, Rome, 00185, Italy
[9]University Hospital of Rome “Tor Vergata”, Rome, 00133, Italy
[10]Department of Oncology, University of Milan, Milan, 20122, Italy

*claudia.alteri@uniroma2.it
[+] These authors contributed equally to this work

Supplementary Material 1. dN and dS parameters in accordance with the year of diagnosis in 1,152 and 66 HIV-1 B subtype *V3* sequences belonging to newly diagnosed and recently infected patients, respectively.

**Year of diagnosis**	***V3* new diagnoses (N=1,152)**				***V3* recent infections (N=66)**			
	**dN, mean±SE**	**P-value**	**dS, mean±SE**	**P-value**	**dN, mean±SE**	**P-value**	**dS, mean±SE**	**P-value**
2007-2009	0.070±0.004		0.117±0.008		0.058±0.009		0.089±0.015	
2010-2012	0.076±0.001	0.0006	0.127±0.003	0.056	0.067±0.005	0.311	0.135±0.016	0.456
2013-2016	0.086±0.002		0.139±0.005		0.071±0.024		0.120±0.020	

dN and dS parameters were calculated by comparing sequences with HIV-1 Consensus B by SNAP. Statistically significant differences were assessed by Kruskal-Wallis test, corrected for Benjamini-Hochberg method.
SE: standard error.

Supplementary Material 2. Prevalence of each CTL escape mutation in 3,328 (A) and 210 (B) HIV-1 B subtype *pol* sequences, belonging to newly diagnosed and recently infected patients, respectively, divided into 4 time frames in accordance with year of diagnosis. Chi-squared tests for trend were used to estimate significant changes over the four time periods.

A

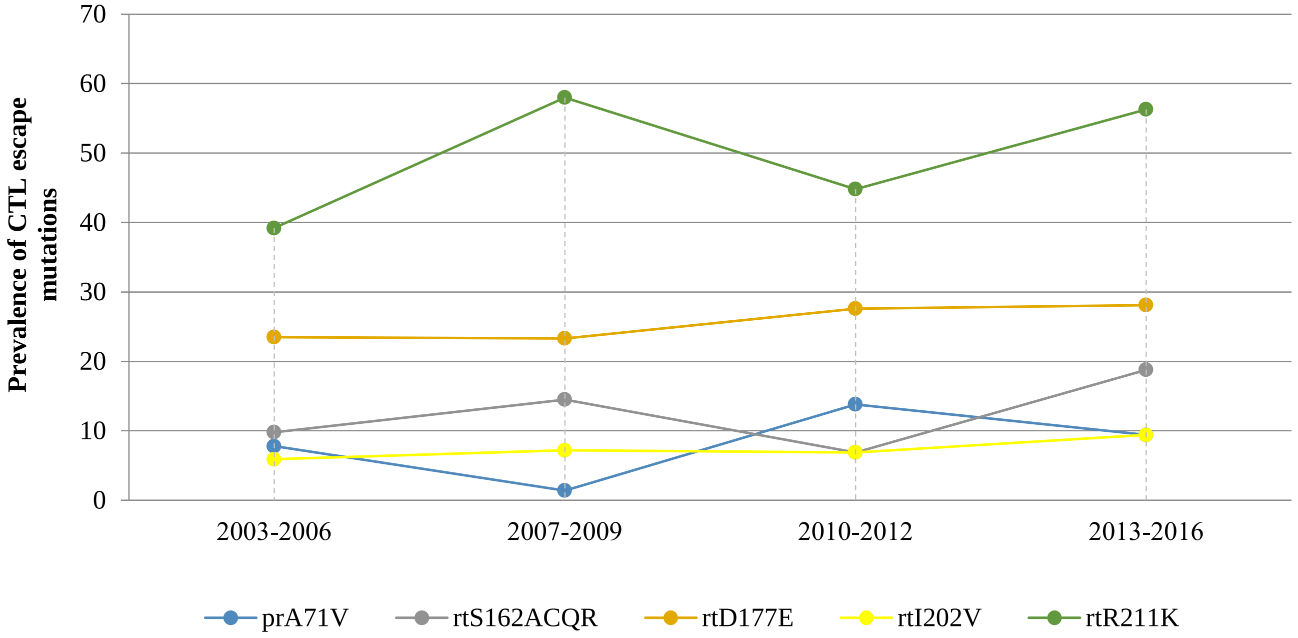

**P-value**	<0.0001	0.0014	0.0014	<0.0001	0.018

B

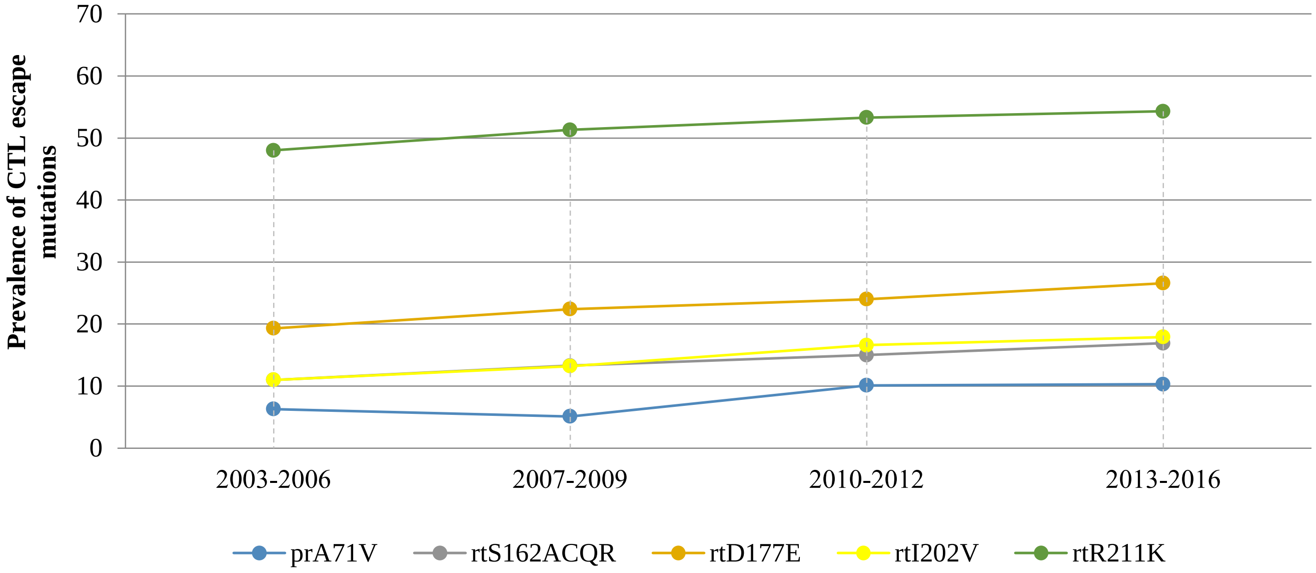

**P-value**	0.237	0.591	0.521	0.609	0.350

Supplementary Material 3. List of CTL escape mutations in *pol* and *V3* sequences retrieved from Los Alamos Immunology Database list

Amino acidic mutation	Location
L10I	*Protease*
K20R	*Protease*
E35D	*Protease*
M46I	*Protease*
I47A	*Protease*
I47V	*Protease*
G48V	*Protease*
I50V	*Protease*
I54V	*Protease*
L63S	*Protease*
A71V	*Protease*
V75I	*Protease*
V82A	*Protease*
V82F	*Protease*
I84V	*Protease*
E6D	*Reverse Transcriptase*
K11R	*Reverse Transcriptase*
K20E	*Reverse Transcriptase*
K20R	*Reverse Transcriptase*
V35I	*Reverse Transcriptase*
A129V	*Reverse Transcriptase*
I132V	*Reverse Transcriptase*
I135L	*Reverse Transcriptase*
I135M	*Reverse Transcriptase*
I135R	*Reverse Transcriptase*
I135T	*Reverse Transcriptase*
I135V	*Reverse Transcriptase*
E138G	*Reverse Transcriptase*
T139I	*Reverse Transcriptase*
V142I	*Reverse Transcriptase*
A158P	*Reverse Transcriptase*
A158T	*Reverse Transcriptase*
I159A	*Reverse Transcriptase*
I159S	*Reverse Transcriptase*
F160I	*Reverse Transcriptase*
F160L	*Reverse Transcriptase*
F160S	*Reverse Transcriptase*
Q161F	*Reverse Transcriptase*
Q161L	*Reverse Transcriptase*
S162A	*Reverse Transcriptase*
S162C	*Reverse Transcriptase*
S162Q	*Reverse Transcriptase*
S162R	*Reverse Transcriptase*
M164S	*Reverse Transcriptase*

T165M	*Reverse Transcriptase*
K166R	*Reverse Transcriptase*
K166T	*Reverse Transcriptase*
E169D	*Reverse Transcriptase*
D177E	*Reverse Transcriptase*
V179E	*Reverse Transcriptase*
V179I	*Reverse Transcriptase*
Y181C	*Reverse Transcriptase*
M184V	*Reverse Transcriptase*
I202V	*Reverse Transcriptase*
Q207E	*Reverse Transcriptase*
Q207G	*Reverse Transcriptase*
R211K	*Reverse Transcriptase*
N6Y	*V3 loop*
T8K	*V3 loop*
S11R	*V3 loop*
I14V	*V3 loop*
G15R	*V3 loop*
Y21V	*V3 loop*
T23I	*V3 loop*
E25Q	*V3 loop*
D29N	*V3 loop*
